# Supplementary figures and images for: Conserved regulatory motifs in the juxtamembrane domain and kinase N-lobe revealed through deep mutational scanning of the MET receptor tyrosine kinase domain
Source: eLife. 2024 Sep 13;12:RP91619. doi: 10.7554/eLife.91619 (PMC11398868; doi:10.7554/eLife.91619)

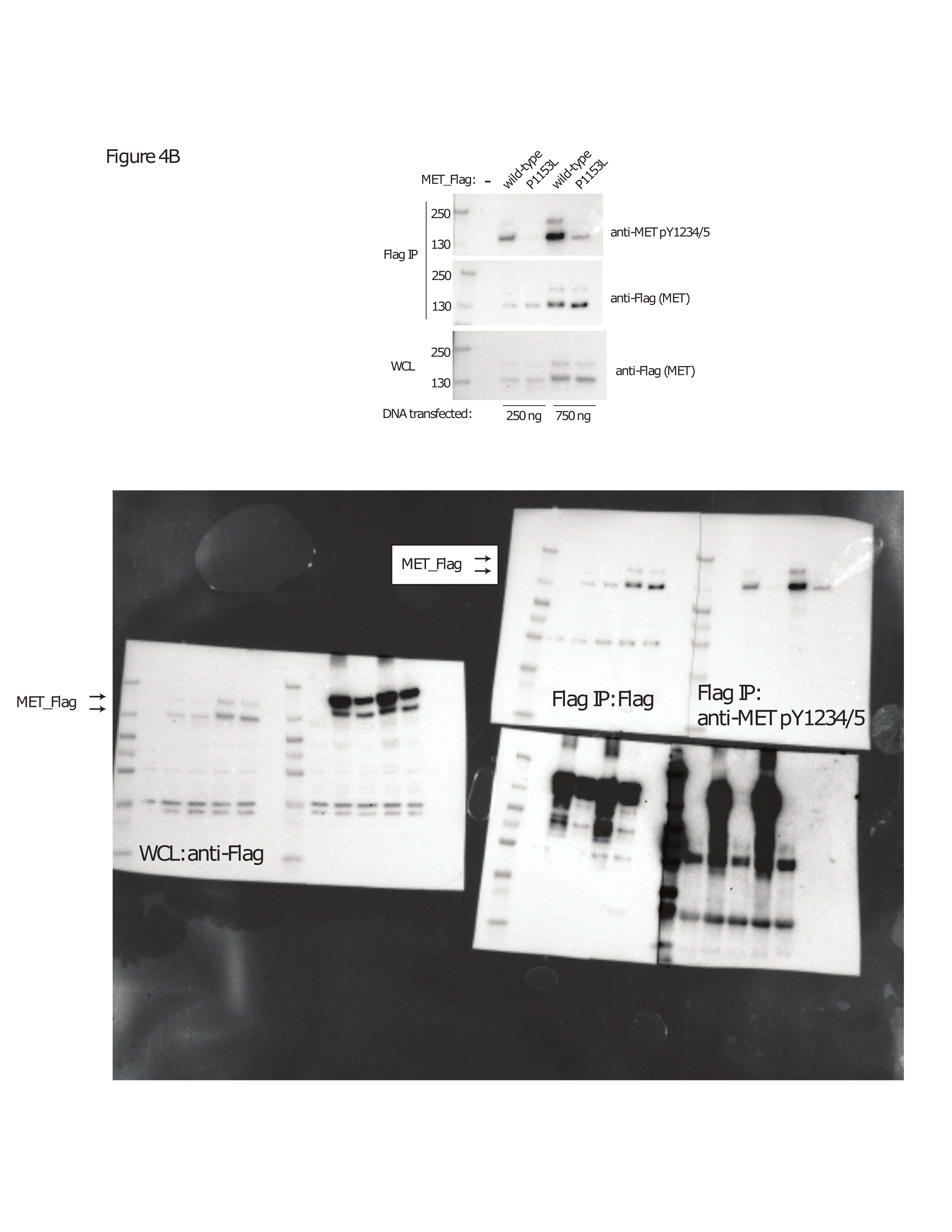

Supplement: Figure 4—source data 1. — Original uncropped whole cell lysate (WCL) and FLAG-IP western blots comparing expression of the wild-type MET receptor to the P1153L mutant (bottom) transfected with 250 ng or 750 ng DNA input, juxtaposed next to the cropped blot (top). [file elife-91619-fig4-data1.zip › Figure 4 - source data 1/Figure 4 - source data 1 Uncropped and labeled blots for figure 4.png]

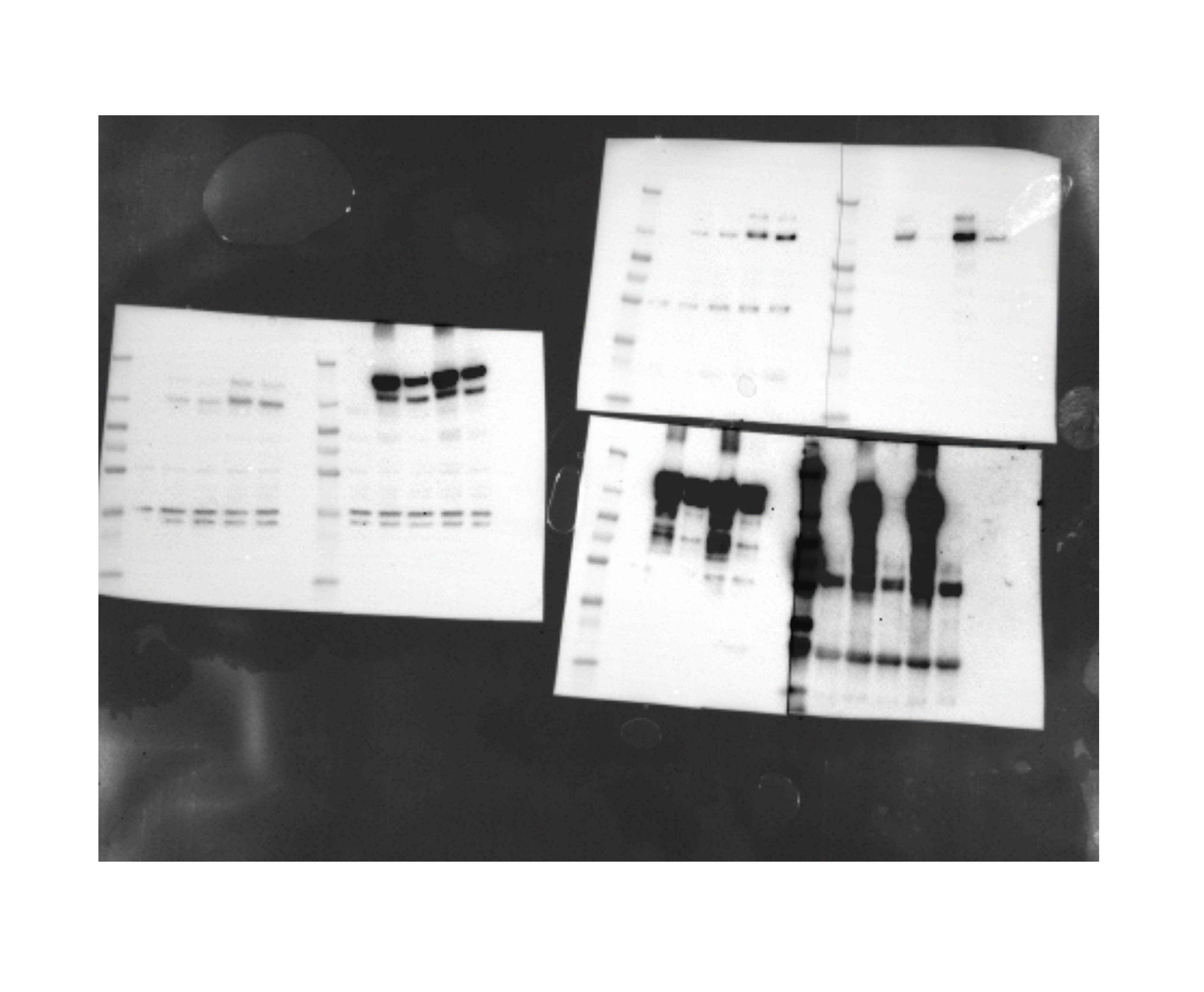

Supplement: Figure 4—source data 2. [file elife-91619-fig4-data2.zip › Figure 4 - source data 2/Figure 4 - source data 2 Raw unedited blots for figure 4.png]

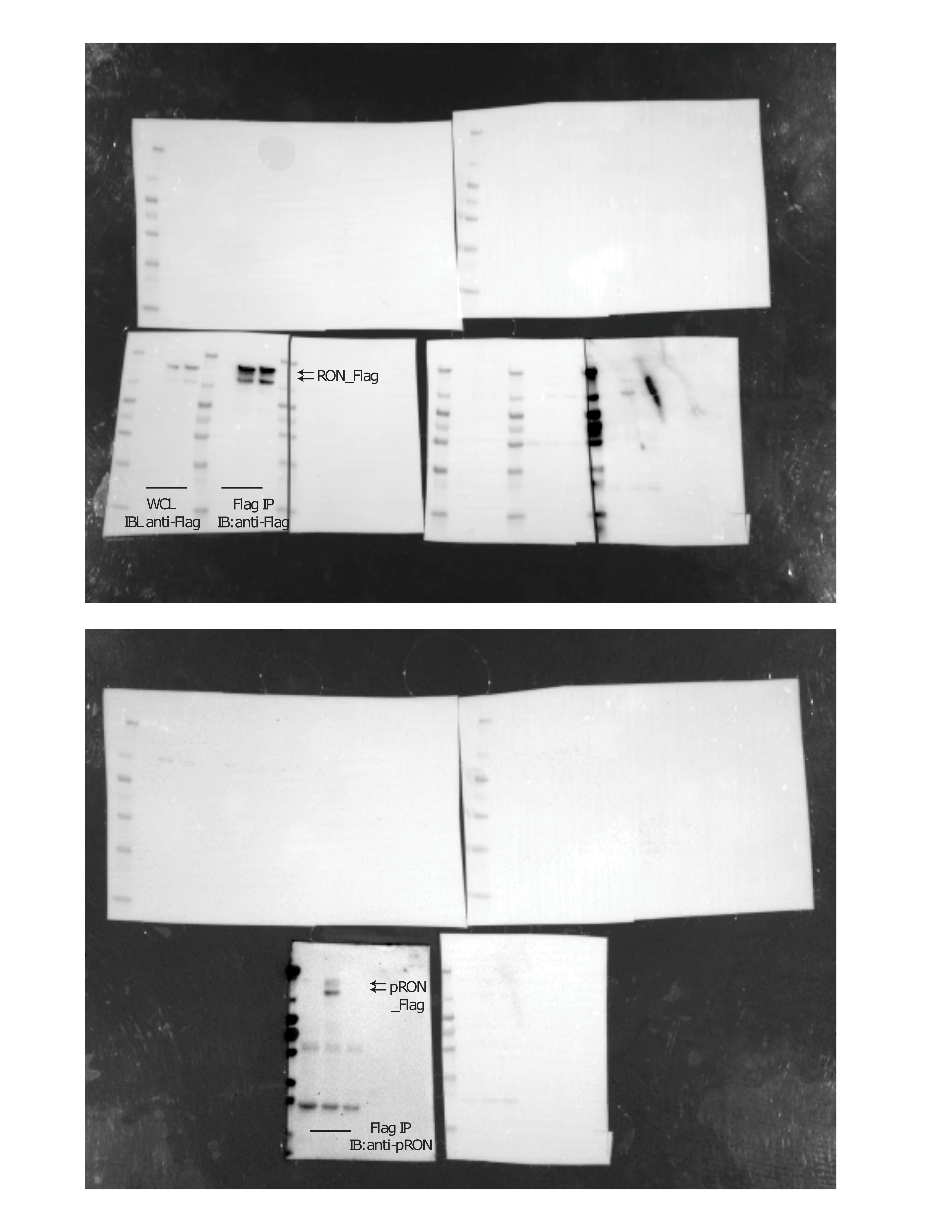

Supplement: Figure 4—figure supplement 1—source data 1. — Original uncropped whole cell lysate (WCL) and FLAG-IP western blots of the wild-type RON receptor against the P1157L RON mutant, transfected with 100 ng or 750 ng DNA input. [file elife-91619-fig4-figsupp1-data1.zip › Figure 4 - figure supplement 1 - source data 1/Figure 4 - figure supplement 1 - source data 1 Uncropped and labeled blots for figure supplement 1.png]

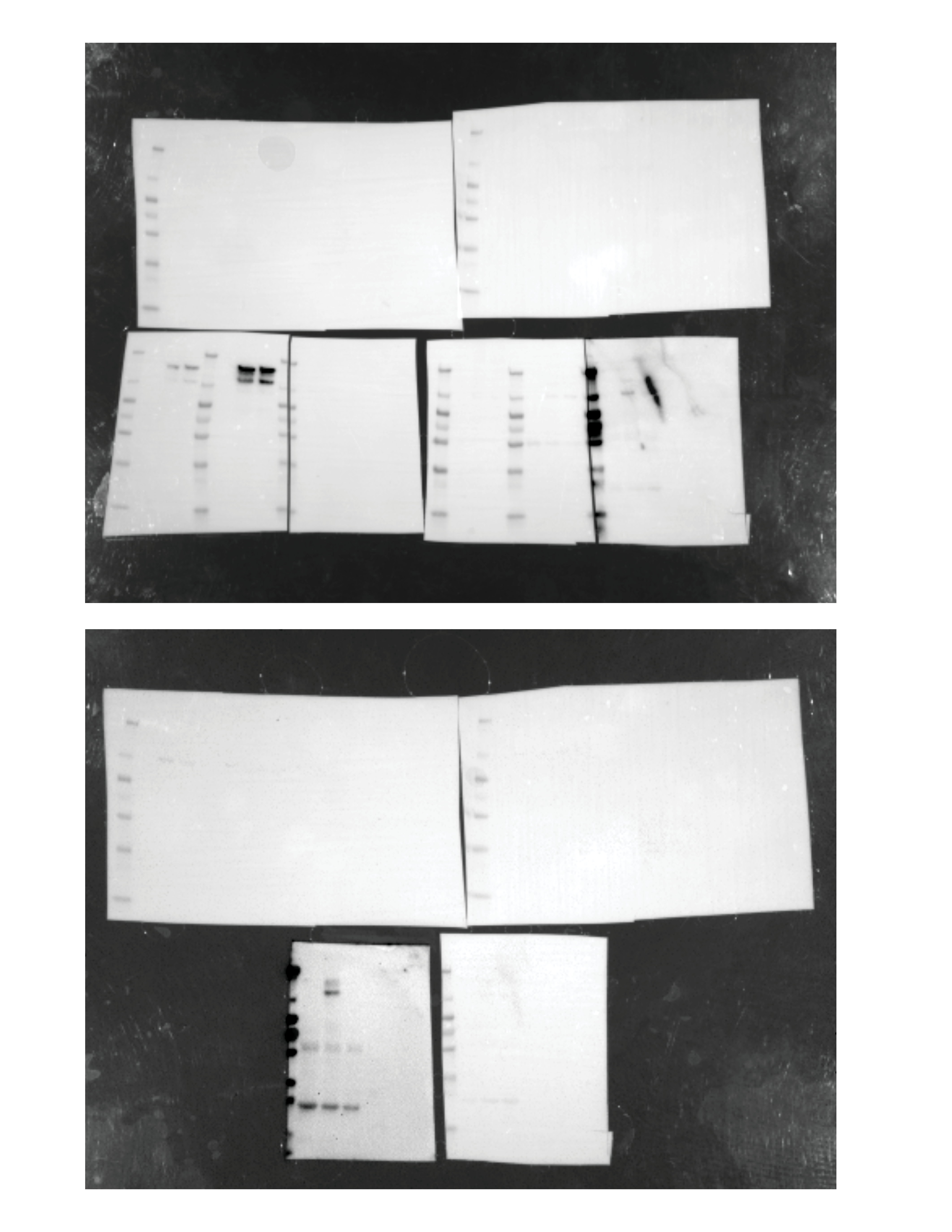

Supplement: Figure 4—figure supplement 1—source data 2. [file elife-91619-fig4-figsupp1-data2.zip › Figure 4 - figure supplement 1 - source data 2/Figure 4 - figure supplement 1 - source data 2 Raw unedited blots for figure supplement 1.png]

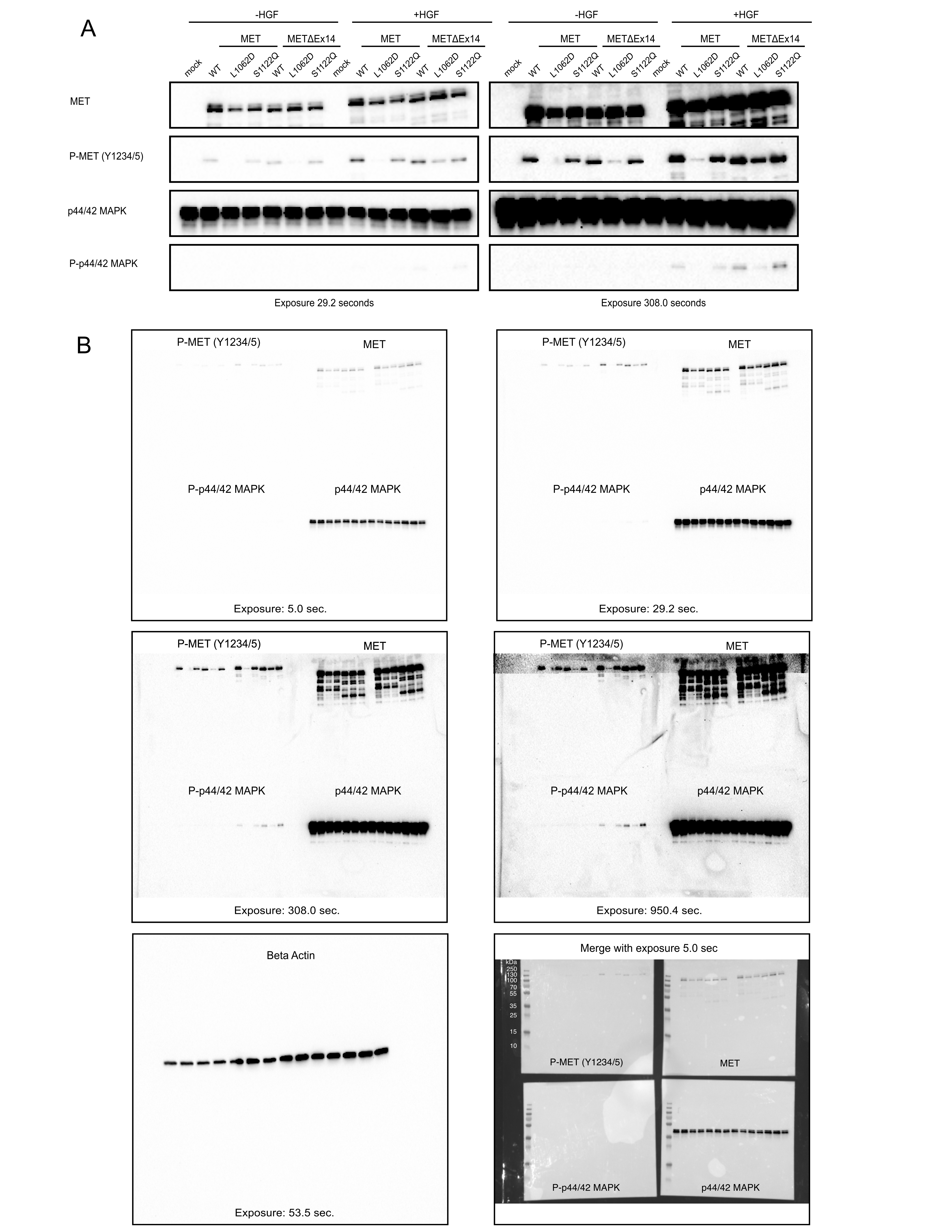

Supplement: Figure 5—source data 1. — (A) Side-by-side comparison of exposures for human MET knockout HeLa cells expressing an FL-MET or FL-METΔEx14 (wild-type, L1062D mutant, and S1122Q mutant) in the presence and absence of HGF (50 ng/ml, 15 min stimulation, 37 °C). (B) Uncropped western blots at varying exposures for each antibody. [file elife-91619-fig5-data1.zip › Figure 5 - source data 1/Figure 5 - source data 1 Uncropped and labeled blots for figure 5..png]

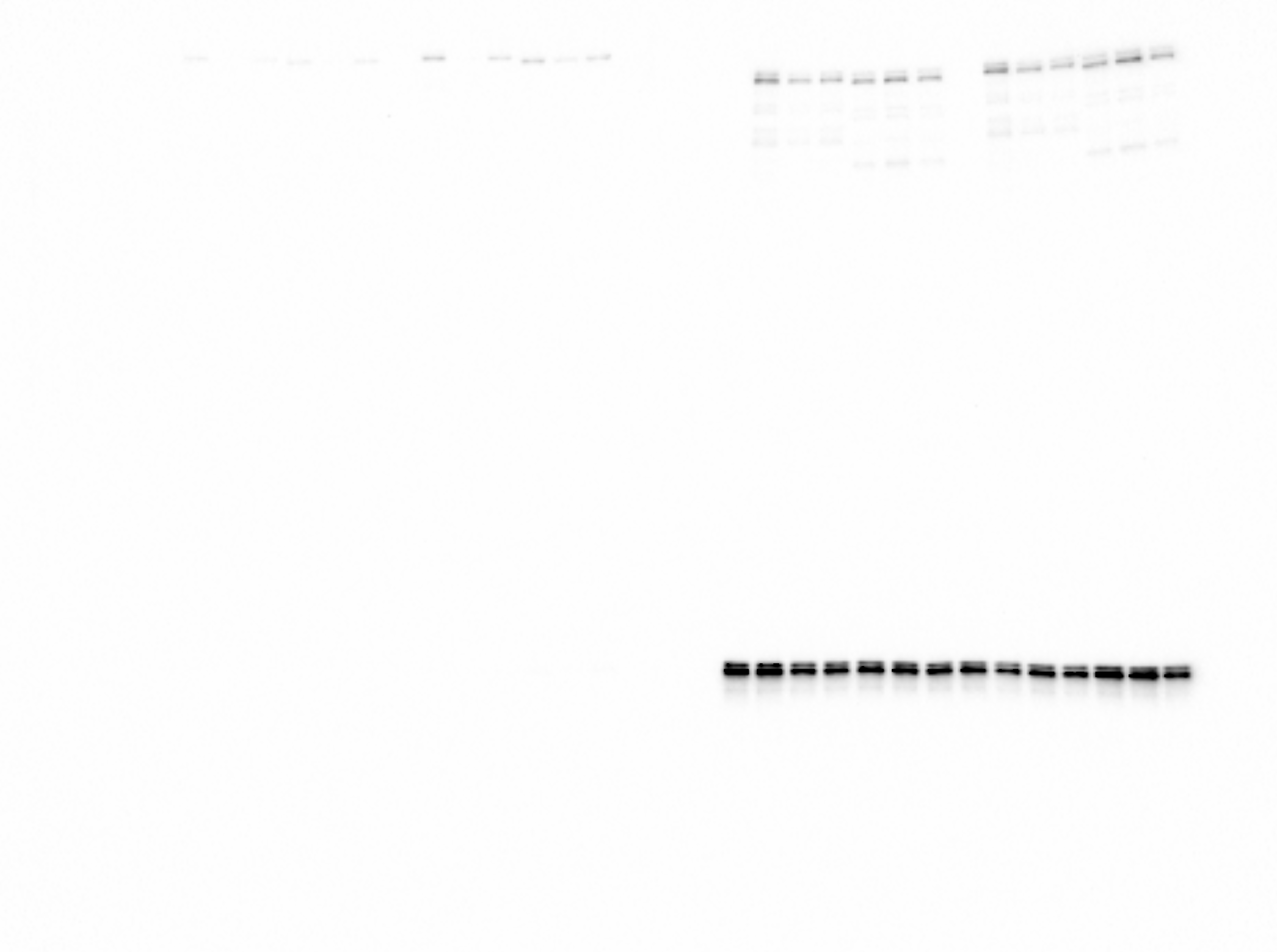

Supplement: Figure 5—source data 2. [file elife-91619-fig5-data2.zip › Figure 5 - source data 2/Figure 5 - source data 1 Raw unedited blots Exposure_5.0sec for figure 5.tif]

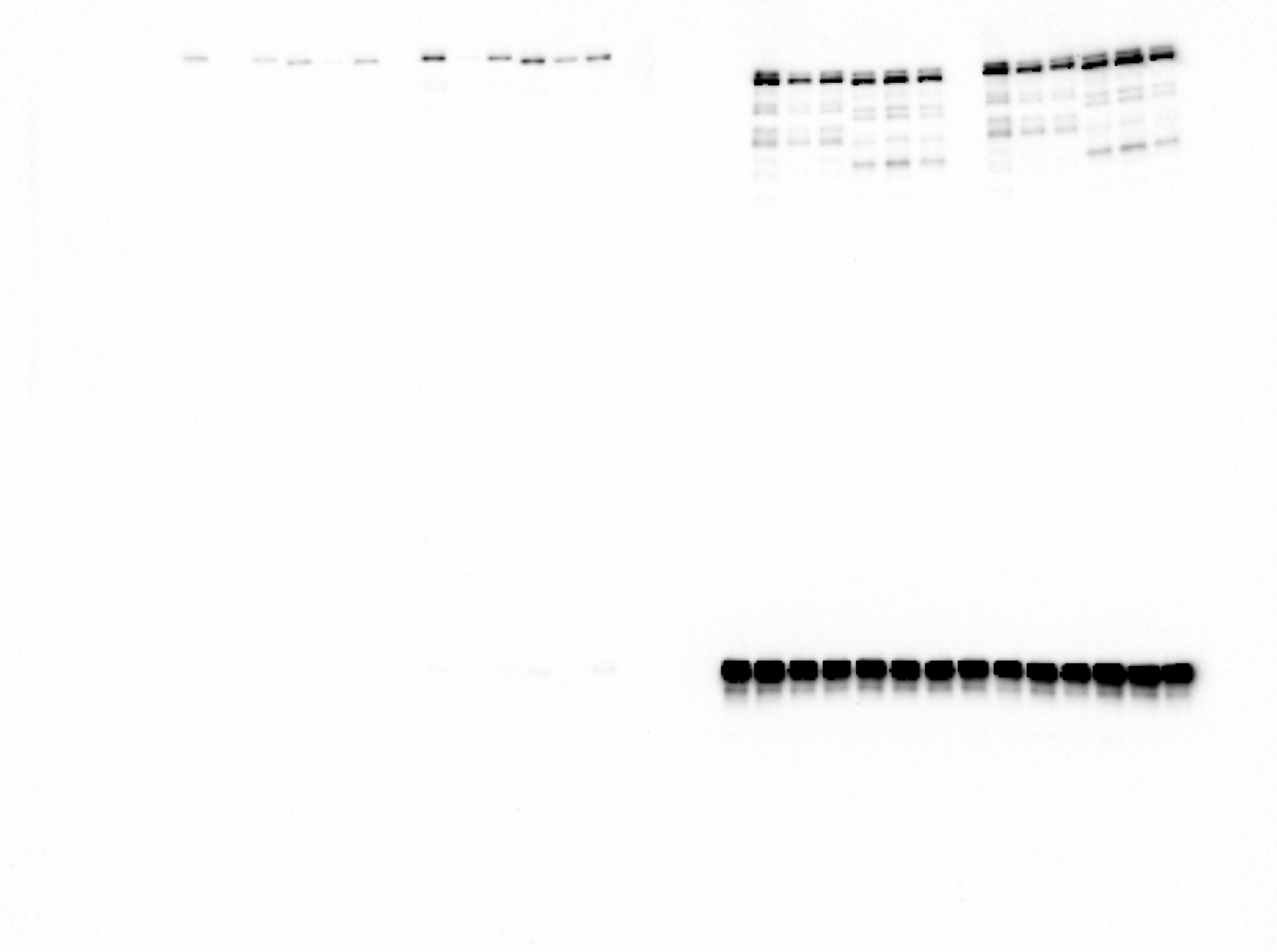

Supplement: Figure 5—source data 2. [file elife-91619-fig5-data2.zip › Figure 5 - source data 2/Figure 5 - source data 2 Raw unedited blots Exposure_29.2sec for figure 5.tif]

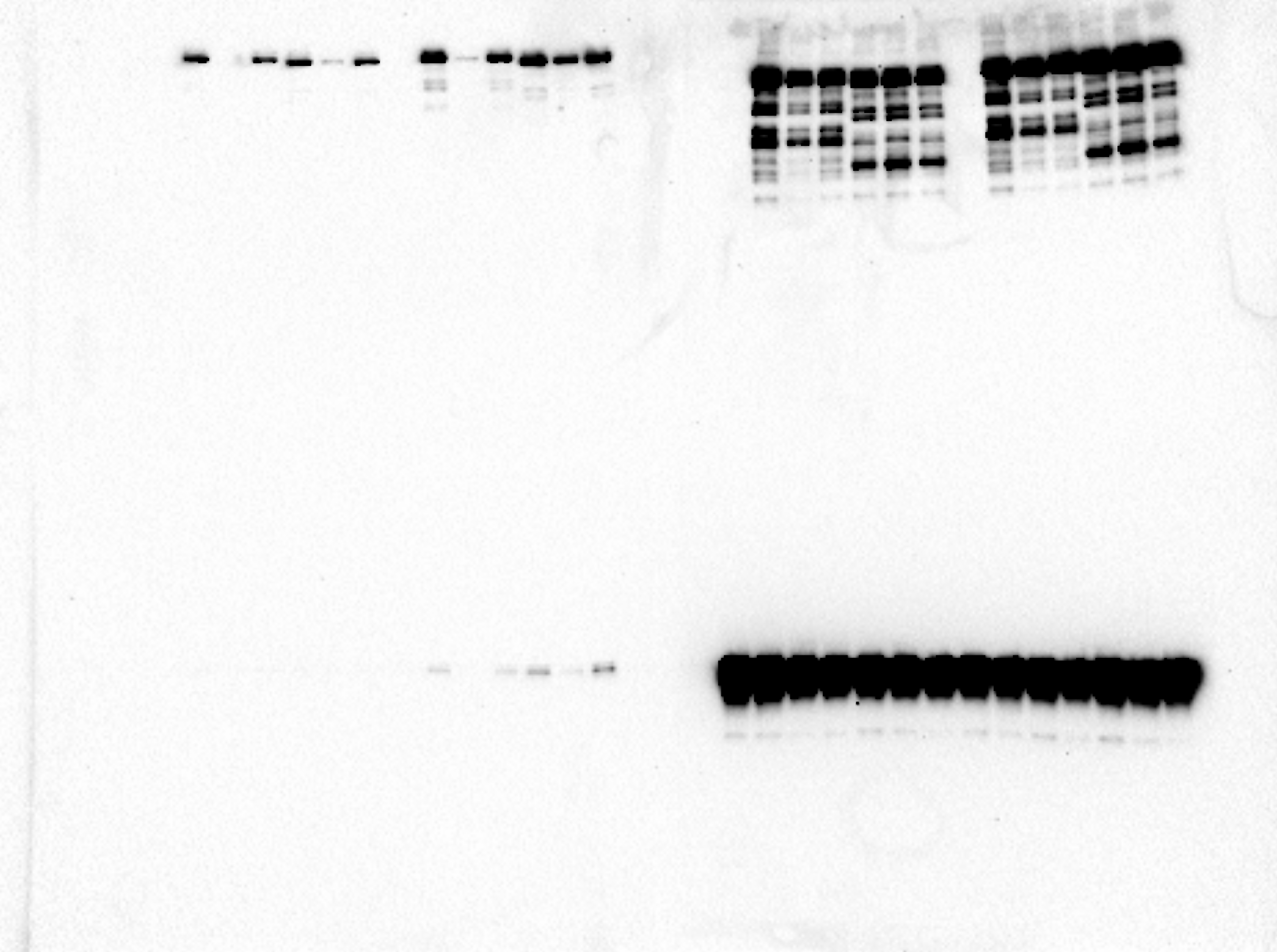

Supplement: Figure 5—source data 2. [file elife-91619-fig5-data2.zip › Figure 5 - source data 2/Figure 5 - source data 3 Raw unedited blots Exposure_308.0sec for figure 5.tif]

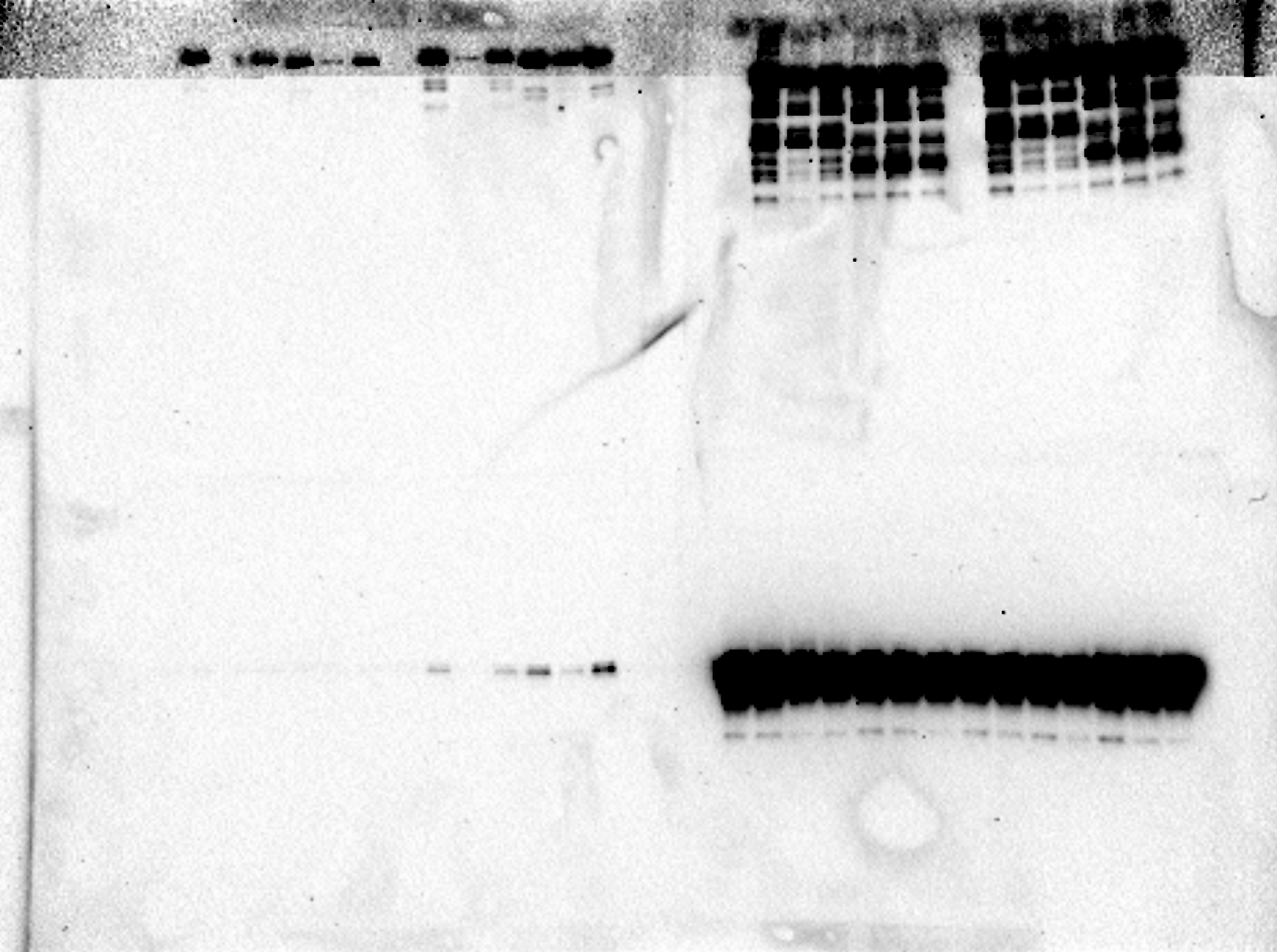

Supplement: Figure 5—source data 2. [file elife-91619-fig5-data2.zip › Figure 5 - source data 2/Figure 5 - source data 4 Raw unedited blots Exposure_950.4sec for figure 5.tif]

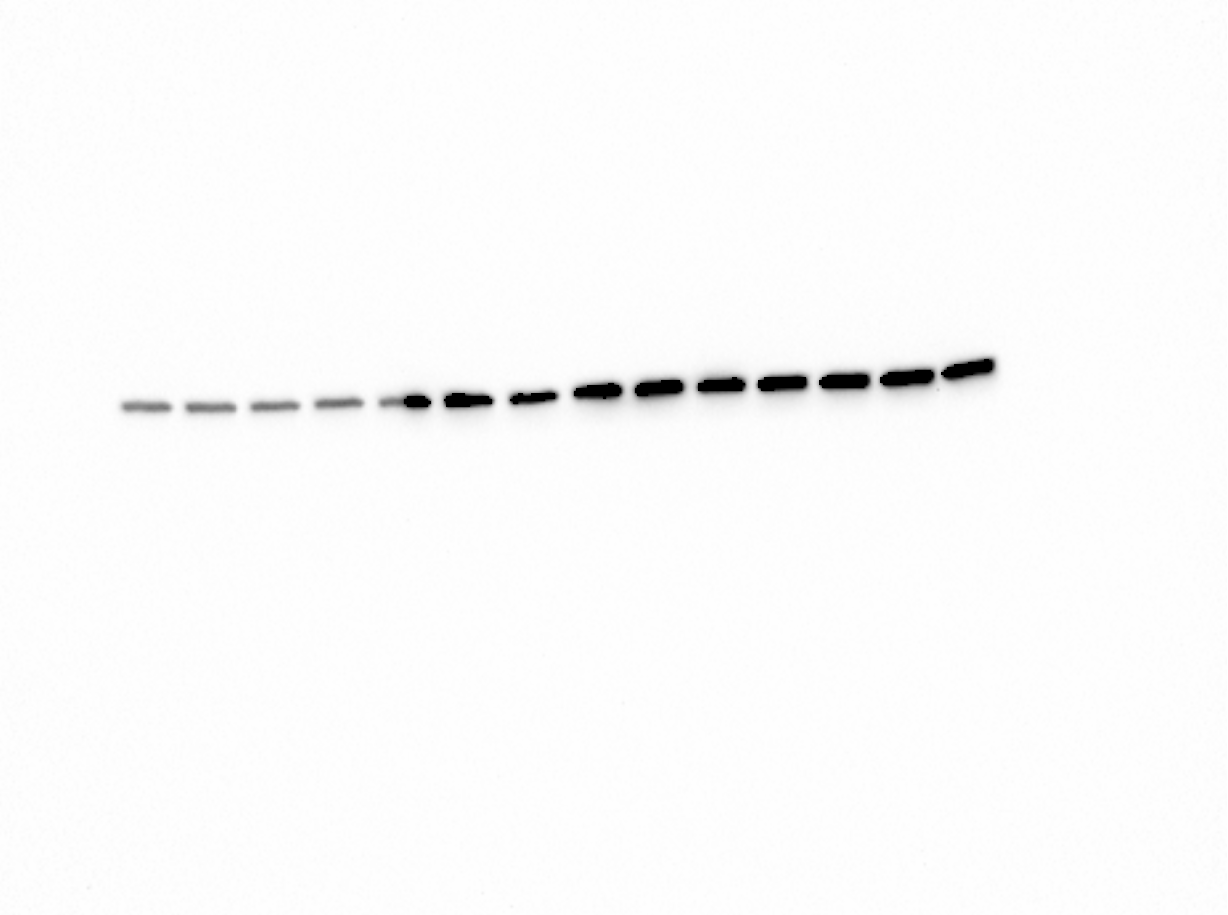

Supplement: Figure 5—source data 2. [file elife-91619-fig5-data2.zip › Figure 5 - source data 2/Figure 5 - source data 5 Raw unedited blots Exposure_29.2sec for figure 5.tif]

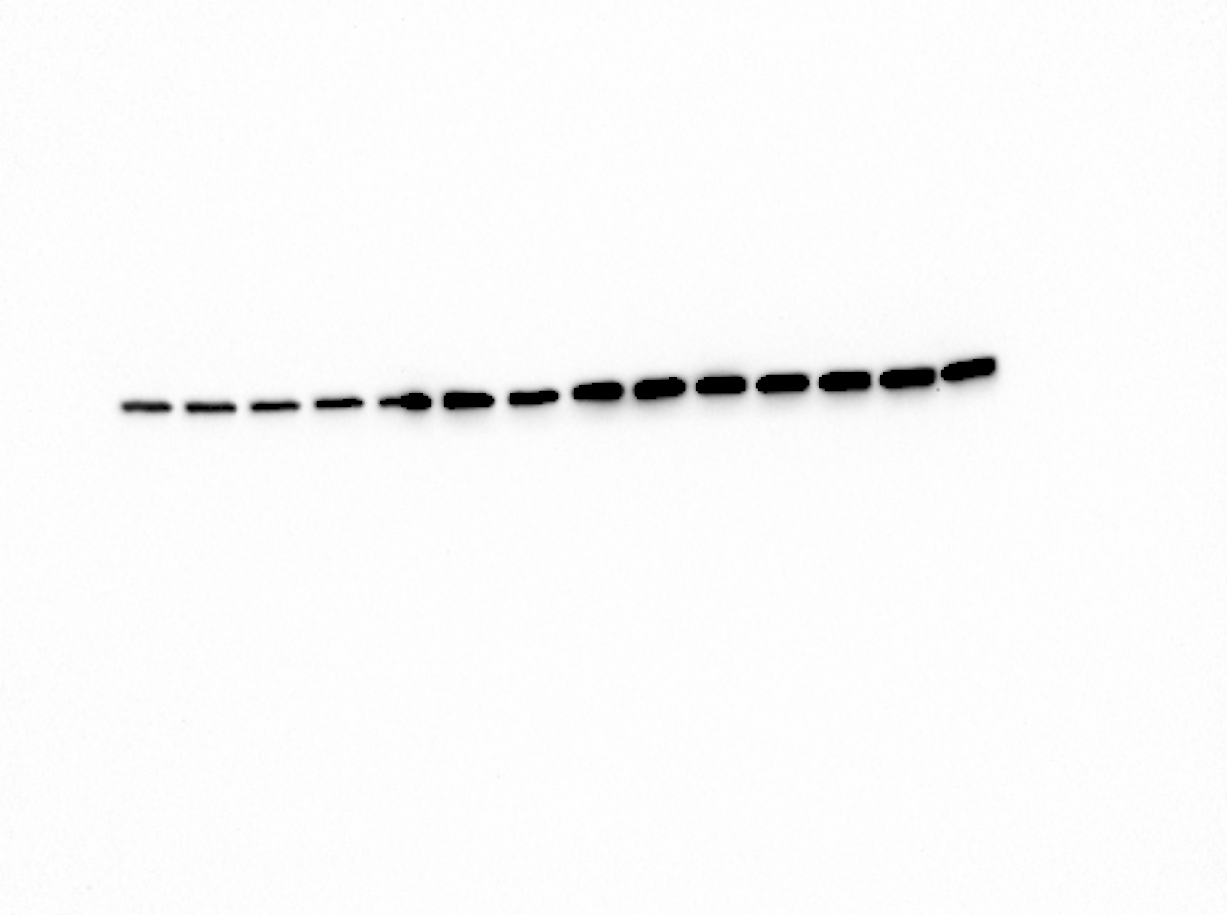

Supplement: Figure 5—source data 2. [file elife-91619-fig5-data2.zip › Figure 5 - source data 2/Figure 5 - source data 6 Raw unedited blots Exposure_53.5sec for figure 5.tif]

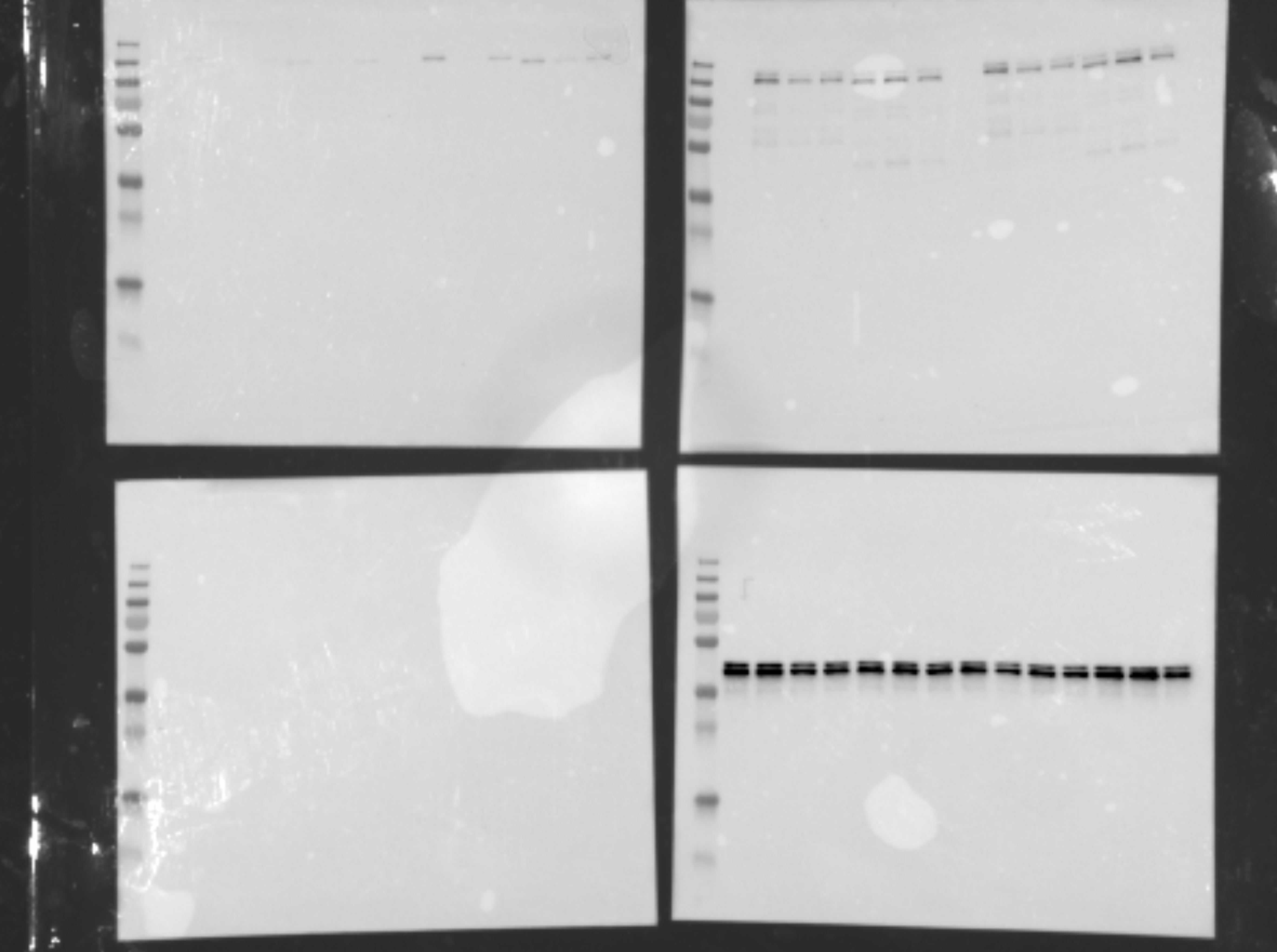

Supplement: Figure 5—source data 2. [file elife-91619-fig5-data2.zip › Figure 5 - source data 2/Figure 5 - source data 7 Raw unedited blots merge_scan for figure 5.tif]

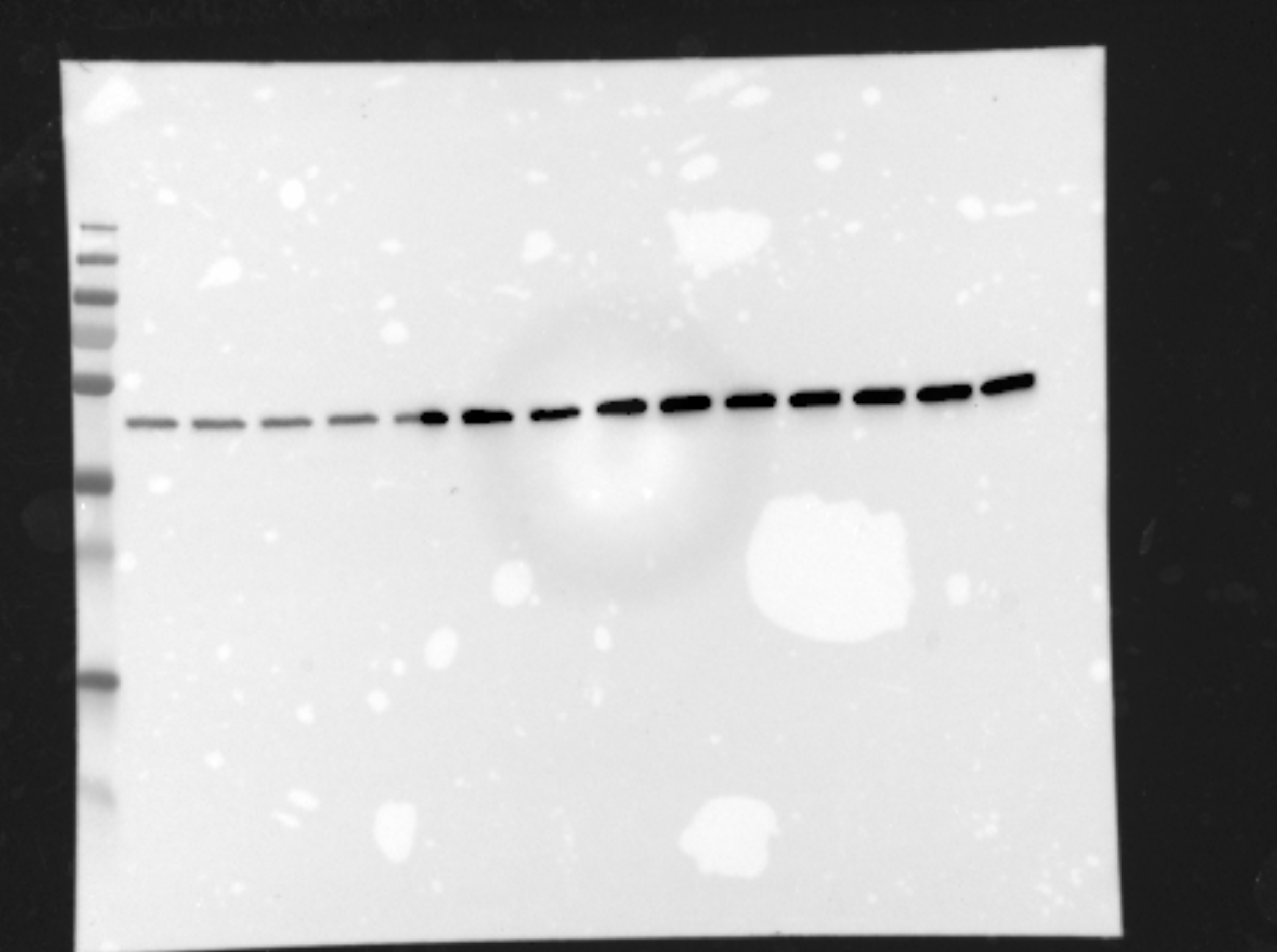

Supplement: Figure 5—source data 2. [file elife-91619-fig5-data2.zip › Figure 5 - source data 2/Figure 5 - source data 8 Raw unedited blots merge_scan for figure 5.tif]
