# Supplementary material for: Conserved regulatory motifs in the juxtamembrane domain and kinase N-lobe revealed through deep mutational scanning of the MET receptor tyrosine kinase domain
Source: eLife. 2024 Sep 13;12:RP91619. doi: 10.7554/eLife.91619 (PMC11398868; doi:10.7554/eLife.91619)
Supplement: MDAR checklist [file elife-91619-mdarchecklist1.docx]

**Materials Design Analysis Reporting (MDAR)**

**Checklist for Authors**

The [MDAR framework](https://osf.io/xfpn4/) establishes a minimum set of requirements in transparent reporting mainly applicable to studies in the life sciences.

*eLife* asks authors to **provide detailed information within their article** to facilitate the interpretation and replication of their work. Authors can also upload supporting materials to comply with relevant reporting guidelines for health-related research (see [EQUATOR Network](http://www.equator-network.org/%20)), life science research (see the [BioSharing Information Resource](http://biosharing.org/)), or animal research (see the [ARRIVE Guidelines](http://www.plosbiology.org/article/info:doi/10.1371/journal.pbio.1000412) and the [STRANGE Framework](https://doi.org/10.1038/d41586-020-01751-5); for details, see *eLife*’s [Journal Policies](https://reviewer.elifesciences.org/author-guide/journal-policies)). Where applicable, authors should refer to any relevant reporting standards materials in this form.

For all that apply, please note **where in the article** the information is provided. Please note that we also collect information about data availability and ethics in the submission form.

**Materials:**

| **Newly created materials** | **Indicate where provided: section/figure legend** | **N/A** |
| --- | --- | --- |
| The manuscript includes a dedicated "materials availability statement" providing transparent disclosure about availability of newly created materials including details on how materials can be accessed and describing any restrictions on access. |  | N/A |
|  |  |  |
| **Antibodies** | **Indicate where provided: section/figure legend** | **N/A** |
| For commercial reagents, provide supplier name, catalogue number and [RRID](https://scicrunch.org/resources), if available. | Details are provided in the materials and methods section under “Validation of variants in the MET and METΔEx14 receptor by western blot”.  Applies to Figure 4B, Figure 4 – figure supplement 4B, and Figure 5F.  All antibodies were purchased from Cell Signaling Technologies. MET pY1234/5 (Cat# 3077), Met (Cat# 8198), P-p44/42 MAPK Erk1/2 (T202/Y204) (Cat# 4376), p44/42 MAPK Erk1/2 (Cat# 4695), β-Actin (Cat# 4970). |  |
|  |  |  |
| **DNA and RNA sequences** | **Indicate where provided: section/figure legend** | **N/A** |
| Short novel DNA or RNA including primers, probes: Sequences should be included or deposited in a public repository. | Details available under “Code and data availability”  Raw next generation sequencing files have been deposited at the NCBI SRA (bioproject PRJNA993160) |  |
|  |  |  |
| **Cell materials** | **Indicate where provided: section/figure legend** | **N/A** |
| Cell lines: Provide species information, strain. Provide accession number in repository OR supplier name, catalog number, clone number, OR RRID. | Materials and Methods section under “Mammalian cell culturing”  Ba/F3 cells were purchased from DSMZ (Cat. ACC 300)  Human MET knockout HeLa cells were purchased from Abcam (Cat. ab265961)  Plat-E cells were gifted by Dr. Wendell Lim (UCSF) |  |
| Primary cultures: Provide species, strain, sex of origin, genetic modification status. |  | N/A |
|  |  |  |
| **Experimental animals** | **Indicate where provided: section/figure legend** | **N/A** |
| Laboratory animals or Model organisms: Provide species, strain, sex, age, genetic modification status. Provide accession number in repository OR supplier name, catalog number, clone number, OR RRID. |  | N/A |
| Animal observed in or captured from the field: Provide species, sex, and age where possible. |  | N/A |
|  |  |  |
| **Plants and microbes** | **Indicate where provided: section/figure legend** | **N/A** |
| Plants: provide species and strain, ecotype and cultivar where relevant, unique accession number if available, and source (including location for collected wild specimens). |  | N/A |
| Microbes: provide species and strain, unique accession number if available, and source. |  | N/A |
|  |  |  |
| **Human research participants** | **Indicate where provided: section/figure legend) or state if these demographics were not collected** | **N/A** |
| If collected and within the bounds of privacy constraints report on age, sex, gender and ethnicity for all study participants. |  | N/A |

**Design:**

| **Study protocol** | **Indicate where provided: section/figure legend** | **N/A** |
| --- | --- | --- |
| If the study protocol has been pre-registered, provide DOI. For clinical trials, provide the trial registration number OR cite DOI. |  | N/A |
|  |  |  |
| **Laboratory protocol** | **Indicate where provided: section/figure legend** | **N/A** |
| Provide DOI OR other citation details if detailed step-by-step protocols are available. |  | N/A |
|  |  |  |
| **Experimental study design (statistics details) *** | | |
| **For in vivo studies: State whether and how the following have been done** | **Indicate where provided: section/figure legend. If it could have been done, but was not, write “not done”** | **N/A** |
| Sample size determination |  | N/A |
| Randomisation |  | N/A |
| Blinding |  | N/A |
| Inclusion/exclusion criteria |  | N/A |
|  |  |  |
| **Sample definition and in-laboratory replication** | **Indicate where provided: section/figure legend** | **N/A** |
| State number of times the experiment was replicated in the laboratory. | Materials and Methods section under “DMS time point selection and sample preparation” |  |
| Define whether data describe technical or biological replicates. | Biological |  |
|  |  |  |
| **Ethics** | **Indicate where provided: section/submission form** | **N/A** |
| Studies involving human participants: State details of authority granting ethics approval (IRB or equivalent committee(s), provide reference number for approval. |  | N/A |
| Studies involving experimental animals: State details of authority granting ethics approval (IRB or equivalent committee(s), provide reference number for approval. |  | N/A |
| Studies involving specimen and field samples: State if relevant permits obtained, provide details of authority approving study; if none were required, explain why. |  | N/A |
|  |  |  |
| **Dual Use Research of Concern (DURC)** | **Indicate where provided: section/submission form** | **N/A** |
| If study is subject to dual use research of concern regulations, state the authority granting approval and reference number for the regulatory approval. |  | N/A |

**Analysis:**

| **Attrition** | **Indicate where provided: section/figure legend** | **N/A** |
| --- | --- | --- |
| Describe whether exclusion criteria were pre-established. Report if sample or data points were omitted from analysis. If yes, report if this was due to attrition or intentional exclusion and provide justification. |  | N/A |
|  |  |  |
| **Statistics** | **Indicate where provided: section/figure legend** | **N/A** |
| Describe statistical tests used and justify choice of tests. | Materials and Methods section under “MET kinase domain variant analysis and scoring” and  “Statistical filtering and classification of mutations” |  |
|  |  |  |
| **Data availability** | **Indicate where provided: section/submission form** | **N/A** |
| For newly created and reused datasets, the manuscript includes a data availability statement that provides details for access (or notes restrictions on access). | Statement of source data availability is provided in the section “Code and data availability” |  |
| When newly created datasets are publicly available, provide accession number in repository OR DOI and licensing details where available. | Source data is publicly available at <https://github.com/fraser-lab/MET_KinaseDomain_DMS> |  |
| If reused data is publicly available provide accession number in repository OR DOI, OR URL, OR citation. | Details are provided in the “Materials and method” section.  Cancer mutations from clinical reports were obtained from cBioPortal (https://www.cbioportal.org/)  RTK protein sequences used in alignments were acquired from The UniProt Consortium (https://www.uniprot.org/) for human MET(uniprot ID P08581)  Mutational mapping and ensemble analysis was applied to crystal structures attained from the PDB (<https://www.rcsb.org/>). Their PDB IDs are referenced throughout the study and provided below.  1R0P 1R1W 2G15 2RFN 2RFS 2WD1 2WGJ 2WKM 3A4P 3CCN 3CD8 3CE3 3CTH 3CTJ 3DKC 3DKF 3DKG 3EFJ 3EFK 3F66 3F82 3I5N 3L8V 3LQ8 3Q6U 3Q6W 3QTI 3R7O 3RHK 3U6H 3U6I 3VW8 3ZBX 3ZC5 3ZCL 3ZXZ 3ZZE 4AOI 4AP7 4DEG 4DEH 4DEI 4EEV 4GG5 4GG7 4IWD 4KNB 4MXC 4R1V 4R1Y 4XMO 4XYF 5DG5 5EOB 5EYC 5EYD 5HLW 5HNI 5HO6 5HOA 5HOR 5HTI 5T3Q 5UAB 5UAD 5YA5 6SD9 6SDC 6SDD 6SDE 6UBW 7B3Z 7B3Q 7B42 7B41 7B44 7B43 7B3T 7B3W 7B3V 7B40 7V3R 7V3S 7Y4T 7Y4U 8GVJ 8AN8 8ANS 8OUU 8OUV 8OU7 8OVZ 8OW3 8OWG |  |
|  |  |  |
| **Code availability** | **Indicate where provided: section/figure legend** | **N/A** |
| For any computer code/software/mathematical algorithms essential for replicating the main findings of the study, whether newly generated or re-used, the manuscript includes a data availability statement that provides details for access or notes restrictions. | Statement of code availability is provided in the section “Code and data availability” |  |
| Where newly generated code is publicly available, provide accession number in repository, OR DOI OR URL and licensing details where available. State any restrictions on code availability or accessibility. | Source code is publicly available at <https://github.com/fraser-lab/MET_KinaseDomain_DMS> |  |
| If reused code is publicly available provide accession number in repository OR DOI OR URL, OR citation. | Details are provided in the “Materials and method” section.  Enrich2 code was used for variant scoring, and original source code is available at https://github.com/FowlerLab/Enrich2/  Structural mapping and structural bioinformatics of this study used tools from Bio3D (http://thegrantlab.org/bio3d/) |  |

**Reporting:**

The MDAR framework recommends adoption of discipline-specific guidelines, established and endorsed through community initiatives.

| **Adherence to community standards** | **Indicate where provided: section/figure legend** | **N/A** |
| --- | --- | --- |
| State if relevant guidelines (e.g., ICMJE, MIBBI, ARRIVE, STRANGE) have been followed, and whether a checklist (e.g., CONSORT, PRISMA, ARRIVE) is provided with the manuscript. |  | N/A |

* We provide the following guidance regarding transparent reporting and statistics; we also refer authors to [Ten common statistical mistakes to watch out for when writing or reviewing a manuscript](https://doi.org/10.7554/eLife.48175).

**Sample-size estimation**

- You should state whether an appropriate sample size was computed when the study was being designed
- You should state the statistical method of sample size computation and any required assumptions
- If no explicit power analysis was used, you should describe how you decided what sample (replicate) size (number) to use

**Replicates**

- You should report how often each experiment was performed
- You should include a definition of biological versus technical replication
- The data obtained should be provided and sufficient information should be provided to indicate the number of independent biological and/or technical replicates
- If you encountered any outliers, you should describe how these were handled
- Criteria for exclusion/inclusion of data should be clearly stated
- High-throughput sequence data should be uploaded before submission, with a private link for reviewers provided (these are available from both GEO and ArrayExpress)

**Statistical reporting**

- Statistical analysis methods should be described and justified
- Raw data should be presented in figures whenever informative to do so (typically when N per group is less than 10)
- For each experiment, you should identify the statistical tests used, exact values of N, definitions of center, methods of multiple test correction, and dispersion and precision measures (e.g., mean, median, SD, SEM, confidence intervals; and, for the major substantive results, a measure of effect size (e.g., Pearson's r, Cohen's d)
- Report exact p-values wherever possible alongside the summary statistics and 95% confidence intervals. These should be reported for all key questions and not only when the p-value is less than 0.05.

**Group allocation**

- Indicate how samples were allocated into experimental groups (in the case of clinical studies, please specify allocation to treatment method); if randomization was used, please also state if restricted randomization was applied
- Indicate if masking was used during group allocation, data collection and/or data analysis
